# P30 Evaluation of the hepatotoxic effect of unreasonable antibiotic therapy among patients in the pre-jaundice period of viral hepatitis A

**DOI:** 10.1093/jacamr/dlae136.034

**Published:** 2024-08-23

**Authors:** Iryna Bondaruk, Tetiana Bevz, Kiarina Myroniuk-Konstantynovych

**Affiliations:** National Pirogov Memorial Medical University, Vinnytsia, Ukraine; National Pirogov Memorial Medical University, Vinnytsia, Ukraine; National Pirogov Memorial Medical University, Vinnytsia, Ukraine

## Abstract

**Background:**

Despite its predominant gastrointestinal localization, the onset of viral hepatitis A (HAV) in most cases is characterized by extra-hepatic manifestations (in 98%—flu-like syndrome in the pre-jaundice period), which complicates timely diagnosis in the primary care, leads to erroneous and unreasonable prescription of antibiotics and therefore creates favourable conditions for formation of antibiotic-resistant strains.

**Patients and methods:**

From the beginning of the outbreak of HAV (Vinnytsia, Ukraine) October–November 2023, 250 patients (64% male, average age 45±8.5 years) were examined for biochemical markers of cytolysis (ALT, AST) and cholestasis (GGT, fractional bilirubin).

**Results:**

A total of 50 examinees (20%) in the pre-jaundice period of HAV indicated antibiotic use (azithromycin, 29 [58%]; amoxicillin/clavulanate, 16 [32%]; cefixime, 5 [10%]). The level of total bilirubin in the blood of all patients amounted to 300–500 μmol/L, the level of ALT was up to 2000–5000 U/L and GGT was 220–260 μmol/L. Among the patients who took antibiotics in the pre-jaundice period, the cytolysis rates were probably higher: ALT 2035.6±166.4 U/L (*P<*0.0001) and AST 1545.5±154.7 U/L (*P<*0.005). Indicators of cholestasis were GGT 254.6±22.2 μmol/L (*P<*0.001), total bilirubin 111.9±8.2 mmol/L (*P<*0.005), direct bilirubin 65.4±5.5 mmol/L (*P<*0.005) and indirect 46.4±5.7 mmol/L (*P<*0.05). Patients who did not take antibiotics in the pre-jaundice period had ALT 2000.6±88.2 U/L, AST 515.5±37.2 U/L, GGT 81.1±12.2μmol/L, total bilirubin 89.9±3.2 mmol/L, direct 43.4±1.5 mmol/L, indirect 33.9±2.2 mmol/L. The duration of jaundice in respondents among those who took antibiotics reliably lasted 14–18 days longer (*P*<0.05).

**Conclusions:**

Errors in the diagnosis of viral diseases lead to inappropriate use of antibacterial drugs, which cause changes in the clinical picture and create prerequisites for a severe course of the disease by combination of the mesenchymal-inflammatory link of the HAV pathogenesis and the hepatotoxic effect of antibacterial therapy.

